# Comparative cytogenetic analysis of some species of the *Dendropsophus microcephalus* group (Anura, Hylidae) in the light of phylogenetic inferences

**DOI:** 10.1186/1471-2156-14-59

**Published:** 2013-07-03

**Authors:** Lilian Ricco Medeiros, Luciana Bolsoni Lourenço, Denise Cerqueira Rossa-Feres, Albertina Pimentel Lima, Gilda Vasconcellos Andrade, Ariovaldo Antonio Giaretta, Gabriel Toselli Barbosa Tabosa Egito, Shirlei Maria Recco-Pimentel

**Affiliations:** 1 Departamento de Biologia Estrutural e Funcional, Instituto de Biologia, Universidade Estadual de Campinas (UNICAMP), 13086-863, Campinas, SP, Brazil; 2 Departamento de Zoologia e Botânica, Instituto de Biociências, Letras e Ciências Exatas, Universidade Estadual Paulista (UNESP), 15054-000, São José do Rio Preto, São Paulo, Brazil; 3 Coordenadoria de Pesquisas em Ecologia, Instituto Nacional de Pesquisas da Amazônia (INPA), 69011-970, Manaus, AM, Brazil; 4 Departamento de Biologia, Centro de Ciências Biológicas e da Saúde, Universidade Federal do Maranhão (UFMA), Campus do Bacanga, 65080-040, São Luís, MA, Brazil; 5 Laboratório de Anuros Neotropicais, Faculdade de Ciências Integradas do Pontal, Universidade Federal de Uberlândia, 38304-402, Ituiutaba, MG, Brazil; 6 Departamento de Polícia Federal, Ministério da Justiça, 68908-901, Macapá, AP, Brazil

**Keywords:** Chromosome, Phylogeny, *Dendropsophus*, Anura

## Abstract

**Background:**

*Dendropsophus* is a monophyletic anuran genus with a diploid number of 30 chromosomes as an important synapomorphy. However, the internal phylogenetic relationships of this genus are poorly understood. Interestingly, an intriguing interspecific variation in the telocentric chromosome number has been useful in species identification. To address certain uncertainties related to one of the species groups of *Dendropsophus*, the *D. microcephalus* group, we carried out a cytogenetic analysis combined with phylogenetic inferences based on mitochondrial sequences, which aimed to aid in the analysis of chromosomal characters. Populations of *Dendropsophus nanus*, *Dendropsophus walfordi*, *Dendropsophus sanborni*, *Dendropsophus jimi* and *Dendropsophus elianeae*, ranging from the extreme south to the north of Brazil, were cytogenetically compared. A mitochondrial region of the ribosomal 12S gene from these populations, as well as from 30 other species of *Dendropsophus*, was used for the phylogenetic inferences. Phylogenetic relationships were inferred using maximum parsimony and Bayesian analyses.

**Results:**

The species *D. nanus* and *D. walfordi* exhibited identical karyotypes (2n = 30; FN = 52), with four pairs of telocentric chromosomes and a NOR located on metacentric chromosome pair 13. In all of the phylogenetic hypotheses, the paraphyly of *D. nanus* and *D. walfordi* was inferred. *D. sanborni* from Botucatu-SP and Torres-RS showed the same karyotype as *D. jimi*, with 5 pairs of telocentric chromosomes (2n = 30; FN = 50) and a terminal NOR in the long arm of the telocentric chromosome pair 12. Despite their karyotypic similarity, these species were not found to compose a monophyletic group. Finally, the phylogenetic and cytogenetic analyses did not cluster the specimens of *D. elianeae* according to their geographical occurrence or recognized morphotypes.

**Conclusions:**

We suggest that a taxonomic revision of the taxa *D. nanus* and *D. walfordi* is quite necessary. We also observe that the number of telocentric chromosomes is useful to distinguish among valid species in some cases, although it is unchanged in species that are not necessarily closely related phylogenetically. Therefore, inferences based on this chromosomal character must be made with caution; a proper evolutionary analysis of the karyotypic variation in *Dendropsophus* depends on further characterization of the telocentric chromosomes found in this group.

## Background

The genus *Dendropsophus* was resurrected by Faivovich *et al.*[1] to include all of the species previously referred to as the 30-chromosome *Hyla* species. However, fewer than 30 of the 92 species of *Dendropsophus* currently known [2] have been karyotyped to date [3-11]. Because the karyotype of *Xenohyla*, which is thought to be the sister group of *Dendropsophus*, is still unknown, it is not possible to infer whether 2n = 30 is a synapomorphy of *Dendropsophus* or *Dendropsophus* + *Xenohyla*[1].

The monophyly of *Dendropsophus* has been supported by molecular data in different studies [1,12,13]. Nevertheless, the monophyly of each of the nine species groups recognized in *Dendropsophus* by Faivovich *et al.*[1] remains an interesting issue for further research.

The *D. microcephalus* group is the most speciose group in the genus, including more than 30 species [1,2]. Wiens *et al.*[12] expanded the number of sampled species of the *D. microcephalus* group and reported the paraphyly of this group with respect to *Dendropsophus riveroi*, which had been allocated to the *D. minimus* group by Duellman [14] and tentatively kept there by Faivovich *et al*. [1]. Fouquet *et al.*[15] recovered within the *D. microcephalus* group not only *D. riveroi* but also *D. gaucheri*, a species previously allocated to the *D. parviceps* group. Despite that the interspecific relationships in the *D. microcephalus* group remain to be elucidated, some of its species were putatively attributed to two clades by Faivovich *et al.*[1]: the *Dendropsophus decipiens* clade (including *D. berthalutzae*, *D. decipiens*, *D. haddadi* and *D. oliveirai*) and the *Dendropsophus rubicundulus* clade (including *D. anataliasiasi*, *D. araguaya*, *D. cachimbo*, *D. cerradensis*, *D. elianeae*, *D. jimi*, *D. rhea*, *D. rubicundulus* and *D. tritaeniatus*). These clades correspond to species groups previously proposed by other researchers [16-18], but Faivovich *et al.*[1] emphasized the absence of a rigorous test for the monophyly of each of these groups.

In addition to the lack of phylogenetic information about the species of the *D. microcephalus* group, several taxonomic questions have persisted. The very small size and highly similar external morphologies of these frogs make their taxonomic identification challenging, resulting in a number of taxonomic problems, including specimen misidentification. For example, the species *D. nanus*, *D. sanborni*, *D. walfordi* and *D. jimi* have been the target of taxonomic discussion [10,15,19]. *Dendropsophus sanborni* was already considered to be a subspecies of *D. nanus*[20,21] but was later considered a valid species by Basso *et al.*[22]. Furthermore, based on morphological data, Lutz [23] and Duellman [24] considered *D. walfordi* to be synonymous with *D. nanus*, but Langone and Basso [19] resurrected *D. walfordi* as a valid species based on vocalization data and tadpole morphology. Recently, Fouquet *et al.*[15], in a phylogenetic analysis that included two specimens of *D. nanus* and one of *D walfordi*, again raised questions regarding these taxa. However, the authors could not draw any conclusions because of the low number of populations sampled and strongly recommended further studies to evaluate the status of *D. nanus* and *D. walfordi*.

Interestingly, the karyotypes found for *D. nanus* and *D. sanborni* specimens by Medeiros *et al.*[10] were the same as those described previously for *D. sanborni*[8] and *D. nanus*[6], respectively. Medeiros *et al.*[10] argued that the morphological similarity of these two species had resulted in their misidentification, which was corroborated by Gruber *et al.*[11], who detected the same karyotypes described by Medeiros *et al.*[10] for *D. nanus* and *D. sanborni*. The karyotypes of *D. walfordi* and *D. jimi* remain unknown.

*Dendropsophus elianeae* is another member of the *D. microcephalus* group with intriguing characteristics that warrant further study. This species was recently described by Napoli and Caramaschi [25] after a revision of specimens previously identified as *D. rubicundulus*. Three additional geographical morphotypes were recognized by Napoli and Caramaschi [25]: one for specimens from the southern regions of the Brazilian states of São Paulo and Minas Gerais, a second for specimens from the northern localities of the Brazilian state of São Paulo and a third morphotype for the specimens from central Brazil. Cytogenetic analyses had previously been performed only with specimens of *D. elianeae* from a locality in the southern region of São Paulo state, and some karyotypic differences between this species and *D. rubicundulus* were described [11]. Cytogenetic and molecular analyses including all three morphotypes described by Napoli and Caramaschi [25] are not yet available.

Considering the interesting variations in the number and size of telocentric chromosomes and the variation in NOR location among the karyotypes of the *Dendropsophus* species [examples in [4,6,10,11]; review in [26], we contributed to the study of the *D. microcephalus* group by (i) describing the karyotypes of *D. walfordi* and *D. jimi* and (ii) providing karyotypic data for several of the populations of *D. nanus, D. sanborni* and *D. elianeae* that were not included in the studies by Skuk and Langone [8], Medeiros el al. [10] or Gruber *et al.*[11]. Additionally, 12S rDNA sequences from all of these species were included in a phylogenetic analysis aiming to better understand the relationships among the species that were analyzed cytogenetically. Because of the taxonomic questions regarding the *D. microcephalus* group, we used the same specimens to obtain DNA sequences and cytogenetic data whenever possible. Through this approach, we also intended to show how analyzing chromosomal characters based on the phylogenetic relationships inferred from another set of data could be helpful in investigating which chromosomal characters constitute synapomorphies, symplesiomorphies and homoplasies. This type of combined analysis could be particularly important when the chromosomal data available are not sufficient for a proper phylogenetic analysis.

## Results

### Phylogenetic analyses

**Figure 1:**
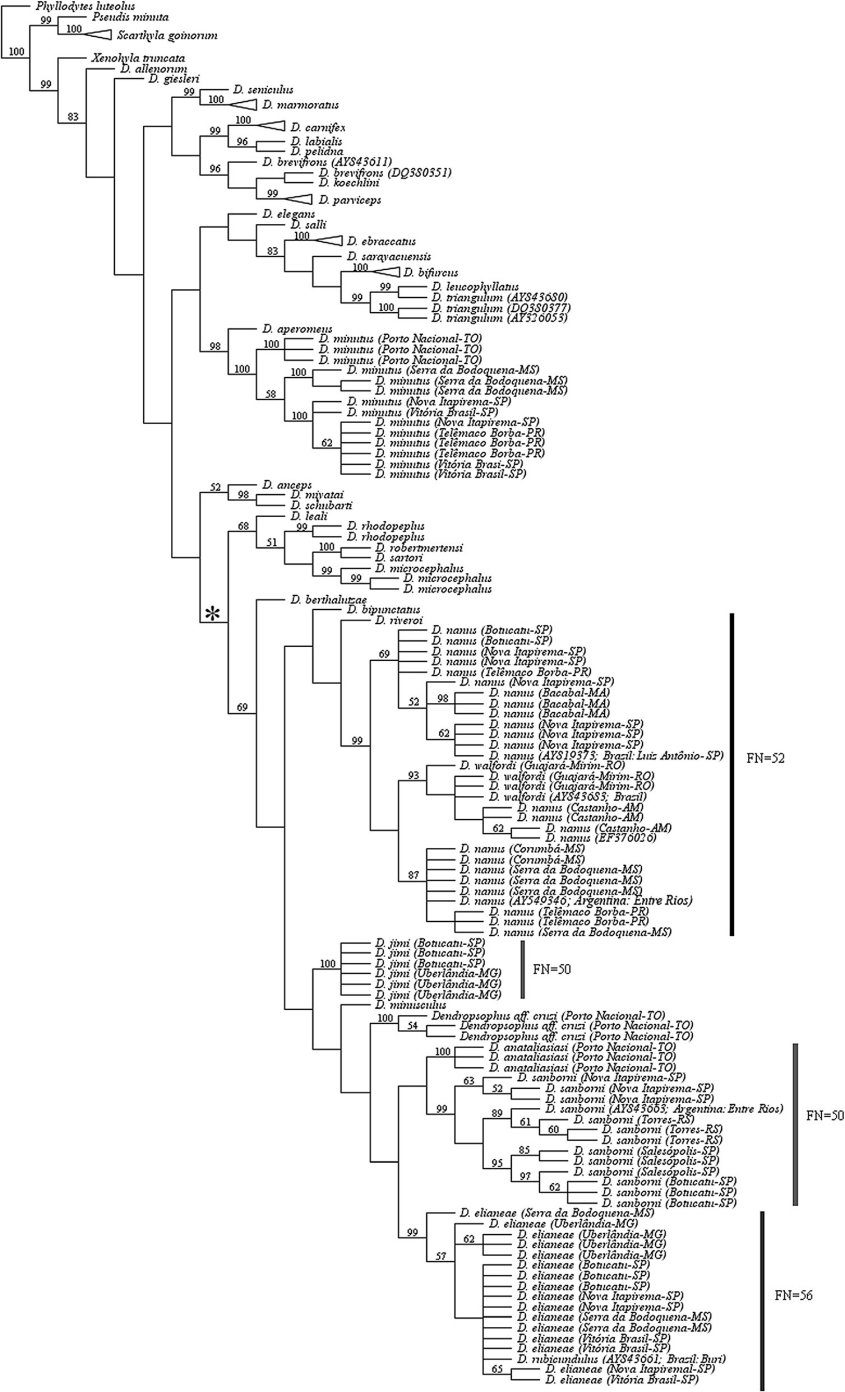
**Strict consensus cladogram scored at 2070 inferred from POY analyses of 12S rDNA sequences.**  Numbers adjacent to nodes indicate bootstrap values. The karyotype fundamental number (FN) is indicated for some species. The asterisk indicates the node of the clade that includes the species of the *D. microcephalus*  group. Sample locations are provided for all the specimens we collected for this study and also for the specimens of *D. nanus*, *D. sanborni*, *D. rubicundulus* and *D. walfordi*  whose 12S rDNA sequences were already available at the GenBank (their GenBank accession numbers are indicated). The GenBank accession numbers are also indicated for *D. brevifrons*  and *D. triangulum*  because these species were not recovered as monophyletic groups.

In all of the inferred topologies (Figure 1 and Additional files 1 and 2: Figures S1 and S2), the species currently assigned to the *D. microcephalus* group included in this study clustered within a clade that also included the species *D. riveroi*. The phylogenetic relationships inferred for *D. jimi*, a species belonging to the *D. microcephalus* group that was not included in the previous phylogenetic analyses of Faivovich *et al.*[1], Wiens *et al.*[12] and Pyron and Wiens [13], differed among the analyses. In the topology inferred through POY analysis, *D. jimi* was the sister group of a clade comprising *D. rubicundulus* (see discussion about the *D. rubicundulus* sequence used below), *D. elianeae*, *D. sanborni*, *D. anataliasiasi*, *Dendropsophus* aff. *cruzi* and *D. minusculus* (Figure 1). However, in the TNT analysis, *D. jimi* and *D. bipunctatus* formed a sister group to the *D. minusculus* + *D. berthalutzae* group (Additional file 1: Figure S1). In the Bayesian analysis, *D. jimi* was included in a polytomy with *D. berthalutzae*, *D. bipunctatus* and *D. minusculus* (Additional file 2: Figure S2).

*Dendropsophus anataliasiasi*, another species not included in the previous studies of phylogenetic relationships, was grouped with *D. sanborni* in the TNT and POY cladograms, but the clade did not receive significant support (Figure 1 and Additional file 1: Figure S1). In the Bayesian analysis, *D. anataliasiasi* was included in a polytomy with *D. sanborni* and *D. elianeae* (Additional file 2: Figure S2).

Interestingly, the sequence assigned to *D. rubicundulus* by Faivovich *et al.*[1] [GenBank: AY843661] clustered among the *D. elianeae* sequences in all of the analyses (Figure 1 and Additional files 1 and 2: Figures S1 and S2). In addition, the specimens of *D. elianeae* were not grouped according to their geographic distribution, despite the fact that several specimens representing the geographic range of this species (central Brazil, Minas Gerais state and different regions of São Paulo state) were analyzed.

Another relevant finding was the paraphyly of *D. nanus* and *D. walfordi*, which was inferred in all the analyses (Figure 1 and Additional files 1 and 2: Figures S1 and S2). Interestingly, the karyotype described for *D. walfordi* was completely indistinguishable from that described for *D. nanus*, as reported below.

### Chromosomal analysis

**Figure 2:**
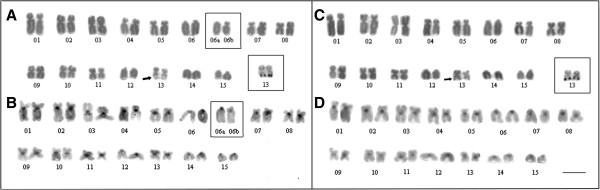
**Karyological data of *Dendropsophus nanus *and *Dendropsophus walfordi. ***Karyotype arranged from Giemsa-stained **(A**, **C)**  and C-banded metaphases **(B**, **D)** of *D. nanus*  **(A**, **B)** and *D. walfordi*  **(C**, **D)**. The insets in **A**  and **C** show the heteromorphic pair 6 of the ZUEC 13179 specimen and the Ag-stained NOR-bearing chromosomes 13. In **B**, note a C-band adjacent to the centromere of chromosome 3 observed in individuals from Telêmaco Borba-PR. The arrows in **A** and **C**  indicate the secondary constrictions of the NORs. Bar = 5 μm.

**Figure 3:**
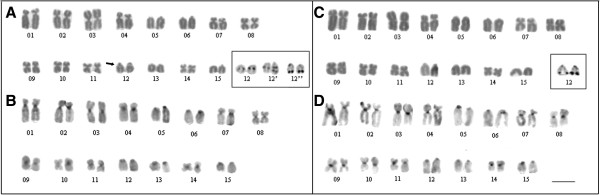
**Karyological data of *Dendropsophus sanborni *and *Dendropsophus jimi. ***Karyotype arranged from Giemsa-stained **(A**, **C)**  and C-banded metaphases **(B**, **D)** of *D. sanborni* **(A**, **B)** and *D. jimi* **(C**, **D)**. The insets in **A** and **C** show the Ag-stained NOR-bearing chromosomes. The inset in **A** shows the NOR-bearing chromosome pairs 12 and 12´, found in Botucatu-SP, and the pair 12´´, found in Torres- RS. The arrow indicates the secondary constriction in the NOR-bearing chromosome pair 12 of *D. sanborni*. Bar = 5 μm.

**Figure 4:**
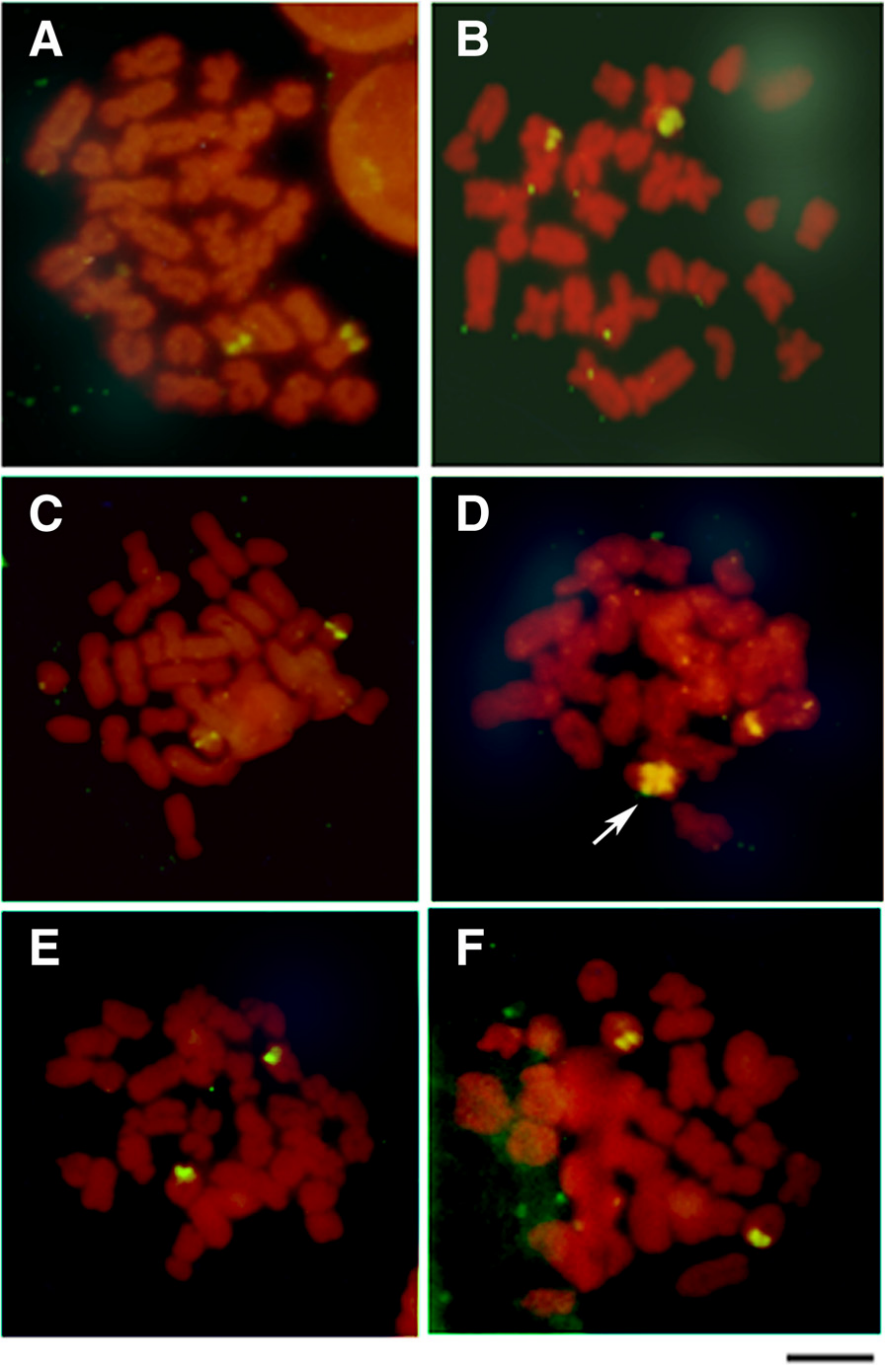
**Chromosome mapping of nucleolar rDNA in *Dendropsophus nanus*, *Dendropsophus walfordi*, *Dendropsophus sanborni *and *Dendropsophus jimi*.** Metaphases of *D. nanus* **(A)**, *D. walfordi* **(B)**, *D. sanborni* from Botucatu-SP **(C**, **D)** and Torres-RS **(E)**, and *D. jimi* **(F)** after FISH with rDNA probes. In **C-E**, note the three different NOR phenotypes found for *D. sanborni*, which are identified by the presence of the NOR-bearing chromosome pairs 12 **(C)**, 12´ **(D)** and 12´´ **(E)** (see text for details). The arrow in **D** indicates the duplicated NOR in one of the homologs of the NOR-bearing chromosome pair 12´. Bar = 5 μm.

**Figure 5:**
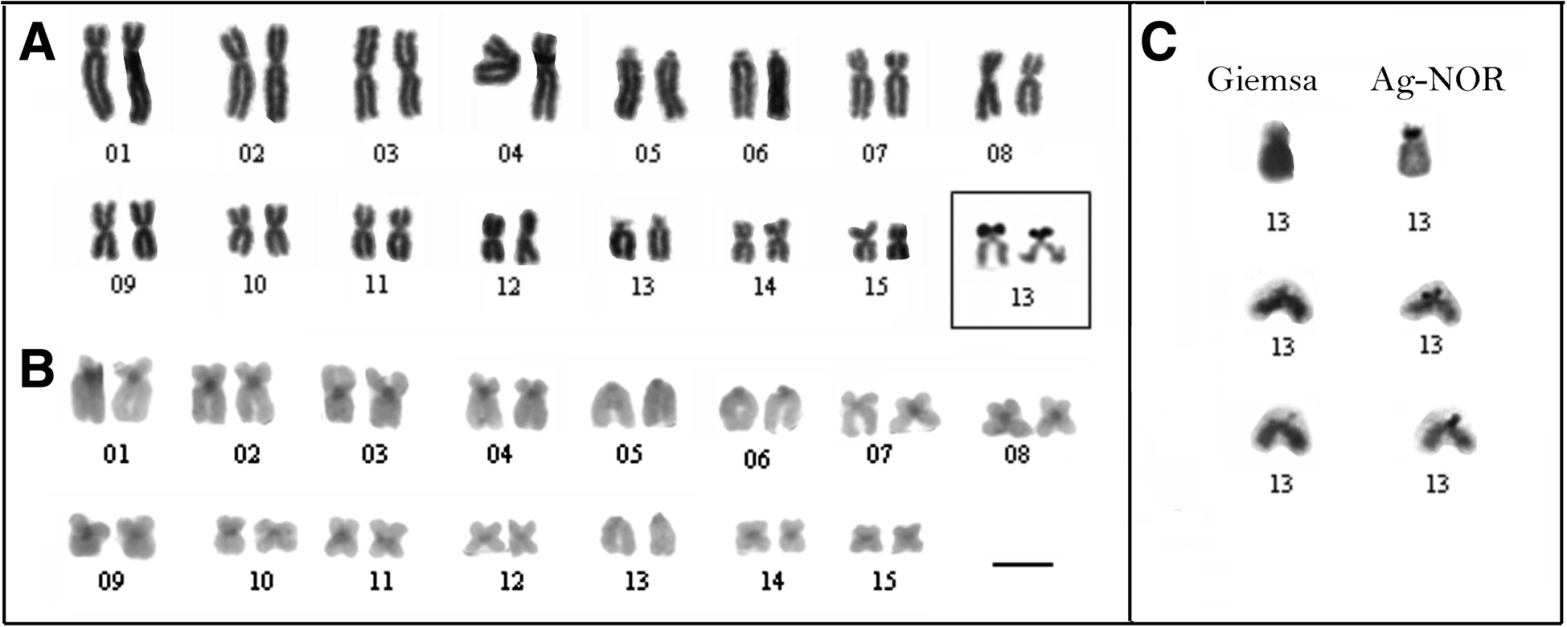
**Karyological data of *Dendropsophus elianeae. ***Karyotype arranged from Giemsa-stained **(A)** and C-banded metaphases **(B)** of *Dendropsophus elianeae*. The inset in **A** shows the Ag-stained NOR-bearing chromosome pair 13. In **C,** NOR-bearing chromosomes of *D. elianeae* after Giemsa staining (left) and sequential Ag-staining (right) are shown. Bar = 5 μm.

*Dendropsophus elianeae*, *D. jimi*, *D. nanus*, *D. sanborni* and *D. walfordi* exhibited karyotypes with 2n = 30 chromosomes (Figures 2, 3, 4 and 5) but differed in the number of telocentric chromosomes. As a proper inference of the homeology of the chromosomes belonging to the different species is not yet possible, we ordered them within each karyotype according to their sizes.

The specimens of *D. nanus* and *D. walfordi* showed very similar karyotypes, with a fundamental number (NF) of 52, 6 pairs of metacentric chromosomes (pairs 3, 8, 9, 10, 11 and 13), 5 pairs of submetacentric chromosomes (pairs 1, 2, 4, 5 and 7) and 4 pairs of telocentric chromosomes (pairs 6, 12, 14 and 15) (Figure 2). One specimen of *D. nanus* from Serra da Bodoquena (ZUEC 13179) presented a karyotype with a heteromorphic pair 6, which was composed of a telocentric morph 6a and a submetacentric morph 6b; its NF was, therefore, 53 (Figures 2A, 2B and Additional file 3: Figure S3). In some *D. nanus* and *D. walfordi* metaphases, a secondary constriction was detected distally on the long arm of pair 13 (Figures 2A and 2C) in the same site detected as a NOR by Ag-NOR staining (Figures 2A and 2C - insets) and by FISH with an rDNA probe (Figures 4A and 4B). The C-banding analysis detected the centromeric regions of all of the chromosomes of *D. nanus* and *D. walfordi* (Figures 2B and 2D). In the C-banded metaphases of specimens of *D. nanus* from Telêmaco Borba, a small pericentromeric C-band was detected in the metacentric chromosome 3 (Figure 2B).

The karyotype of *D. sanborni* was very similar to that of *D. jimi*, with NF = 50, 6 pairs of metacentric chromosomes (pairs 3, 8, 9, 10, 11 and 14), 4 pairs of submetacentric chromosomes (pairs 1, 2, 4 and 7) and 5 pairs of telocentric chromosomes (pairs 5, 6, 12, 13 and 15) (Figure 3). In *D. sanborni*, interindividual variation was observed in relation to NOR sites. Although specimens from Torres-RS provided a NOR that was detected terminally in the long arm of chromosome 12 (pair 12´´ in Figures 3A and 4E), specimens from Botucatu-SP presented a NOR that was detected interstitially in the long arm of chromosome 12 (pair 12 in Figures 3A and 4C). Interestingly, one specimen from Botucatu-SP exhibited a heteromorphic chromosome pair 12 (designated pair 12´) that was composed of a chromosome with an interstitial NOR and a chromosome with an interstitial and a terminal NOR (Figures 3A- inset and 4D). In *D. jimi*, both Ag-NOR staining and FISH with the rDNA probe detected a NOR in the terminal region of the long arm of chromosome 12 (Figures 3C and 4F). C-banding analysis detected the centromeric regions of all of the chromosomes in the *D. jimi* and *D. sanborni* karyotypes (Figures 3B and 3D).

All of the specimens of *D. elianeae* had a karyotype with FN = 56 composed of 8 pairs of metacentric chromosomes (pair 3, 8–12, 14–15), 4 pairs of submetacentric chromosomes (pairs 1, 2, 4 and 7), 1 pair of subtelocentric chromosomes (pair 13) and 2 pairs of telocentric chromosomes (pairs 5 and 6) (Figure 5). The NOR was detected in the short arm of the subtelocentric chromosome 13 by Ag-NOR staining (Figures 5A – inset and 5C). This NOR could be seen as a secondary constriction in only 14 chromosomes of 10 Giemsa-stained metaphases (Figures 5A and 5C) and these samples were used for the inference of the arm ratio of chromosome 13. All eight measured Giemsa-stained metaphases were subjected to analysis using the Ag-NOR method to confirm the NOR location. Because the average value for the arm ratio was 3.32, the NOR-bearing chromosome 13 was classified as subtelocentric, following the convention of Green and Sessions [27]. In the majority of the Giemsa-stained metaphases in which the NOR constriction could not be observed, the limit of the short arm of chromosome 13 could not be identified; as such, it could have been misinterpreted as telocentric. In these cases, sequential staining using Giemsa and the Ag-NOR method was especially useful for the correct recognition of the NOR-bearing chromosome pair. The C-banding analysis detected all of the centromeric regions in the karyotype of this species (Figure 5B).

## Discussion

Our phylogenetic analyses increased the number of sampled species of the *D. microcephalus* group from 11 [the number of species analyzed by Wiens *et al.*[12], not including *D. rubicundulus* because of the taxonomic questions regarding sequence AY843661 mentioned below] to 15 due to the inclusion of *D. anataliasiasi*, *D. cruzi*, *D. elianeae* and *D. jimi*. All of the sampled species from this group clustered in a clade that also included *D. riveroi*, a species that was previously assigned tentatively to the *D. minimus* group [1,14] but has a known phylogenetic relationship with the species of the *D. microcephalus* group, as indicated by Wiens *et al.*[12] and Fouquet *et al.*[15]. Despite the congruence of these data, inference based on a larger number of *Dendropsophus* species is still necessary to conclusively assess the monophyly of the *D. microcephalus* group. The inclusion of a number of species currently assigned to the *D. microcephalus* group, which comprises more than 30 species, is particularly important not only for testing the monophyly of this group but also for proper inferring the internal relationships.

With regard to the *D. rubicundulus* clade, as tentatively defined by Faivovich *et al*. [1], our sample also left questions. In our analyses, the sampled species of this group did not compose a clade. In the POY analysis, the less-inclusive clade that clustered the three sampled species of the *D. rubicundulus* group (*D. anataliasiasi*, *D. elianeae* and *D. jimi*) also included *D. sanborni*, *D. minusculus* and *Dendropsophus* aff. *cruzi*. In the TNT and Bayesian inferences, the *D. rubicundulus* group was additionally paraphyletic with respect to *D. bipunctatus* and *D. bertalutzae*, the latter being the only species of the *D. decipiens* group [1] sampled here. Therefore, the monophylies of the *D. rubicundulus* group and the *D. decipiens* group remain to be tested.

Because the internal relationships of the *D. microcephalus* group are still unclear, it is not yet possible to present an accurate evolutionary interpretation of all of the cytogenetic data available for the *Dendropsophus* species, including the variation of the karyotypic fundamental number. However, some clades were stable and highly supported in all of our inferences and allowed an interesting combined phylogenetic and cytogenetic analysis that provided relevant contributions on the species level. One of these concerns the species *D. nanus* and *D. sanborni*, which were subject of taxonomic controversy until recently. The 12S rDNA sequences we obtained for *D. nanus* were very similar to those assigned to this species by Faivovich *et al.*[1] [GenBank: AY549346] and Wiens *et al.*[28] [GenBank: AY819373]; these sequences were clustered together in our phylogenetic analyses within a clade that did not include *D. sanborni*. The same populations of *D. nanus* and *D. sanborni* sampled for our phylogenetic analyses were also karyotyped here, as well as by Medeiros *et al.*[10], and showed 2n = 30 karyotypes with FN = 52 or 50, respectively. Our combined analyses corroborate the hypothesis proposed by Medeiros *et al.*[10] and supported by Gruber *et al.*[11] regarding the identification of the species analyzed. However, they disagree with the identification presented by Skuk and Langone [8]. This finding highlights how important it is to generate and analyze a different set of data obtained from the same populations or specimens. Therefore, combining other sets of data, in addition to their cytogenetic characters, seems to be an interesting approach that should be considered by cytogeneticists, particularly when studying not only species with known taxonomic questions but also polymorphic species.

All of the specimens of *D. nanus* that were analyzed cytogenetically here (from Telêmaco Borba-PR, Bacabal-MA and Serra da Bodoquena-MS) showed the same karyotype previously described by Medeiros *et al.*[10] (for specimens of *D. nanus* from Nova Itapirema-SP) and Gruber *et al.*[11] (for specimens of *D. nanus* from Rio Claro-SP), with the exception of one specimen from Serra da Bodoquena, which had FN = 53 instead of FN = 52. The heteromorphism observed for pair 6 in this specimen of *D. nanus*, in which chromosome 6a was morphologically similar to the telocentric pair 6 of the other specimens whereas chromosome 6b was submetacentric, might be explained by pericentric inversion. The hypothesis of inversion is based on the chromosomal morphology and consistent total length of the submetacentric chromosome, which would have been altered in the case of a chromosomal deletion or translocation. Another feature found in only a few specimens of *D. nanus* is a heterochromatin block observed close to the centromere in the long arm of chromosome 3 that occurs in some specimens from Telêmaco Borba.

The typical karyotype of *D. nanus*, with FN = 52, differs greatly from that of *D. sanborni*, which has five pairs of telocentric chromosomes (FN = 50). The karyotype of the specimens of *D. sanborni* from Torres-RS described here is identical to that previously presented for specimens from Nova Itapirema-SP [10], even with regard to the terminal location of the NOR in the telocentric chromosome 12. Karyotyping of specimens of *D. sanborni* from Botucatu-SP revealed an intrapopulational variation because three morphs of the NOR-bearing chromosome 12 were found. One of these morphs exhibiting an interstitial NOR was the same type found in the specimens of *D. sanborni* from Rio Claro-SP analyzed by Gruber *et al.*[11].

Interestingly, the karyotype of *D. jimi*, which was described here for the first time, is identical to that of specimens of *D. sanborni* from Nova Itapirema-SP [10], Torres-RS and Botucatu-SP (only those with a terminal NOR in chromosome pair 12). Based on the phylogenetic relationships of *D. jimi* and *D. sanborni* inferred here, the karyological similarities between these taxa cannot be interpreted as synapomorphies of these species. This finding is a clear example of the importance of interpreting chromosomal data in light of their phylogenetic inferences. If this caution had not been taken and only the similarities between the karyotypes of these anurans had been reported, it could lead to misinterpretation by non-specialist readers, and the cytogenetic similarities could be inadequately read as synapomorphies for the species in study.

Another relevant contribution of our analyses concerns the paraphyly of *D. nanus* with respect to *D. walfordi*. The nesting of *D. walfordi* (from Brazil) among three specimens of *D. nanus* (two from French Guiana and one from Argentina) was already recovered by Fouquet *et al.*[15,29]. In our study, which included specimens from seven Brazilian localities, the paraphyly of *D. nanus* and *D. walfordi* was also inferred. Interestingly, the karyotype of *D. walfordi* was indistinguishable from that of *D. nanus* using the classical cytogenetic techniques employed here. Therefore, our data corroborate the proposal of synonymy of *D. walfordi* and *D. nanus* presented by Lutz [23] and Duellman [24]. However, further studies that include other DNA sequences, such as 16S rDNA and COI sequences, and other source of data are still necessary to conduct a proper taxonomic review of this issue.

Finally, our phylogenetic analyses did not cluster the specimens of *D. elianeae* according to their geographical range and no cytogenetic variation was detected among the specimens of *D. elianeae* from Botucatu-SP, Nova Itapirema-SP and Serra da Bodoquena-MS, which are localities that correspond to those of the geographic morphotypes SSP (southern São Paulo), NSP (northern São Paulo) and CBR (central Brazil), respectively, recognized by Napoli and Caramaschi [25]. In addition, it is worth noting that the 12S rDNA sequence of a specimen previously assigned to *D. rubicundulus* [GenBank: AY843661] [1] was clustered among the sequences of *D. elianeae* in all of our phylogenetic analyses. This finding raises doubts regarding the taxonomic identification of that *D. rubicundulus* specimen. Unfortunately, other specimens of *D. rubicundulus* could not be included in our analyses, so further studies are necessary to clarify this issue.

The karyotype reported here for the *D. elianeae* specimens is most likely the same as that previously described by Gruber *et al.*[11] for specimens of *D. elianeae* from the Rio Claro municipality in southern São Paulo state, despite the distinct numeric classification of some chromosome pairs. Because several of the chromosomes in this karyotype are sufficiently similar in terms of size and centromeric position and could not be differentiated by any specific marker (differential C-band, for example), the attribution of a numeric classification to the chromosomes of this karyotype is a difficult task that results in only a tentative arrangement of the chromosome pairs. Therefore, the differences in the numeric classification of the chromosomes between the *D. elianeae* karyotype described by Gruber *et al.*[11] and that presented here most likely do not constitute real cytogenetic differences.

Another apparent difference between the *D. elianeae* karyotype described by Gruber *et al.*[11] and the karyotype presented here involves the morphological classification of the NOR-bearing chromosome. Gruber *et al.*[11] classified this chromosome as telocentric but also reported the presence of a NOR in its short arm. Because we measured all of the NOR-bearing chromosomes in which the secondary constriction of the NOR could be easily identified, we consider this chromosome to be subtelocentric, but it is most likely the same chromosome that carries the NOR in the karyotype described by Gruber *et al.*[11]. In addition, by mapping the karyotype fundamental numbers on the phylogenies (Figures 1, 2), we may consider the karyotype of *D. elianeae* to be derived from an ancestral karyotype with FN = 50 similar to those found in *D. jimi* and *D. sanborni*. Otherwise, neither the homeology between the telocentric chromosomes of the above-mentioned species nor the possible rearrangement that might have resulted in the presumed FN increase can be elucidated.

Therefore, neither the cytogenetic data nor the phylogenetic analyses presented here were able to corroborate the geographic variation reported by Napoli and Caramaschi [24] for the species *D. elianeae*. Further studies including DNA markers that are more informative for this scale of analysis, such as 16S rDNA, COI gene and microsatellite sequence markers, could be very useful in this case.

## Conclusions

In conclusion, we observed that while in some cases the obtained cytogenetic data do not help to distinguish between valid species of *Dendropsophus* (such as *D. jimi* and *D. sanborni*), in others, the number of telocentric chromosomes clearly differs interspecifically, which can be useful in taxonomic analyses (such as for *D. nanus* and *D. sanborni*). Despite the interesting variation in the karyotypic fundamental number found in *Dendropsophus*, it is not yet possible to make a clear inference of homeology between the telocentric chromosomes of different species because several chromosomes of each karyotype have been found to be very similar based on analyses using the most commonly employed cytogenetic techniques. Therefore, the current numerical classifications of the chromosomes in the *Dendropsophus* karyotypes do not reflect interspecific chromosomal homeologies and, in most cases, the inference of homeology between the telocentric chromosomes of different species is merely speculative. Thus, to allow for the formulation of a proposal regarding the chromosomal rearrangements involved in the karyotypic differentiation of the *Dendropsophus* species, further descriptions of new chromosomal markers and elucidation of the phylogeny of this genus are still necessary*.*

The above considerations regarding the homeology of chromosomal characters were only possible because the cytogenetic data were analyzed in the light of phylogenetic inferences. Therefore, this could be an interesting approach to analyze the evolution of chromosomal data, even when the available karyotypes are not sufficient for generating proper phylogenetic inferences by themselves.

## Methods

### Taxon sampling

Specimens of *D. elianeae, D. jimi*, *D. nanus*, *D. sanborni* and *D. walfordi* from localities ranging from the extreme south to the north of Brazil were used in cytogenetic and phylogenetic analyses. The sampled *D. elianeae* individuals included specimens from central Brazil (Serra da Bodoquena-MS), Minas Gerais state (Uberlândia municipality), and southern (Botucatu municipality) and northern (Nova Itapirema and Vitória Brasil municipalities) areas of São Paulo state (Table 1). The phylogenetic analyses also included 12S rDNA sequences from specimens of *D. anataliasiasi* and *D. minutus*. The animals were collected and submitted to euthanasia under permit issued by the Instituto Brasileiro do Meio Ambiente e dos Recursos Naturais Renováveis (IBAMA, processes 10461 and 02001.008876/01-83). The voucher number and collection site of each of the specimens analyzed are indicated in Table 1.

**Table 1:** Specimens included in the analyses

**Taxon**	**Voucher number**	**Sex (M:male; F: female)**	**Locality**	**Cytogenetic analysis**	**GenBank accession number**	**Reference**
D. microcephalus group						
D. anataliasiasi	ZUEC13138	M	Porto Nacional, Tocantins, Brazil	-	JX287452	This work
D. anataliasiasi	ZUEC13139	M	Porto Nacional, Tocantins, Brazil	-	JX287453	This work
D. anataliasiasi	ZUEC13140	M	Porto Nacional, Tocantins, Brazil	-	JX287454	This work
D. berthalutzae	CFBH 5418		Duque de Caxias, RJ, Brazil	-	AY843607	[1] Faivovich et al. (2005)
D. bipunctatus	MRT5946		Serra do Teimoso, Jussari, Bahia, Brazil	-	AY843608	[1] Faivovich et al. (2005)
D. elianeae	ZUEC12455	M	Uberlândia, Minas Gerais, Brazil	-	JX287455	This work
D. elianeae	ZUEC12459	M	Uberlândia, Minas Gerais, Brazil	Yes	-	This work
D. elianeae	ZUEC12460	M	Uberlândia, Minas Gerais, Brazil	Yes	JX287458	This work
D. elianeae	ZUEC13130	M	Uberlândia, Minas Gerais, Brazil	-	JX287456	This work
D. elianeae	ZUEC13131	M	Uberlândia, Minas Gerais, Brazil	-	JX287457	This work
D. elianeae	ZUEC12273	M	Botucatu, São Paulo, Brazil	Yes	-	This work
D. elianeae	ZUEC12274	M	Botucatu, São Paulo, Brazil	Yes	-	This work
D. elianeae	ZUEC12275	M	Botucatu, São Paulo, Brazil	Yes	JX287401	This work
D. elianeae	ZUEC12276	M	Botucatu, São Paulo, Brazil	Yes	JX287402	This work
D. elianeae	ZUEC12277	M	Botucatu, São Paulo, Brazil	Yes	JX287403	This work
D. elianeae	SMRP128.1	M	Botucatu, São Paulo, Brazil	Yes	-	This work
D. elianeae	SMRP128.2	M	Botucatu, São Paulo, Brazil	Yes	-	This work
D. elianeae	SMRP128.3	M	Botucatu, São Paulo, Brazil	Yes	-	This work
D. elianeae	SMRP128.6	M	Nova Itapirema, São Paulo, Brazil	Yes	-	This work
D. elianeae	DZSJRP7964	M	Nova Itapirema, São Paulo, Brazil	Yes	JX287406	This work
D. elianeae	DZSJRP7965	M	Nova Itapirema, São Paulo, Brazil	Yes	JX287405	This work
D. elianeae	DZSJRP7966	M	Nova Itapirema, São Paulo, Brazil	Yes	JX287404	This work
D. elianeae	DZSJRP7967	M	Vitória Brasil, São Paulo, Brazil	-	JX287412	This work
D. elianeae	DZSJRP7968	M	Vitória Brasil, São Paulo, Brazil	-	JX287411	This work
D. elianeae	DZSJRP7969	M	Vitória Brasil, São Paulo, Brazil	-	JX287410	This work
D. elianeae	ZUEC12465	M	Serra da Bodoquena, Mato Grosso do Sul, Brazil	Yes	JX287407	This work
D. elianeae	ZUEC12466	M	Serra da Bodoquena, Mato Grosso do Sul, Brazil	Yes	-	This work
D. elianeae	ZUEC12467	M	Serra da Bodoquena, Mato Grosso do Sul, Brazil	Yes	JX287408	This work
D. elianeae	ZUEC12468	M	Serra da Bodoquena, Mato Grosso do Sul, Brazil	Yes	JX287409	This work
D. elianeae	ZUEC12469	M	Serra da Bodoquena, Mato Grosso do Sul, Brazil	Yes	-	This work
D. elianeae	ZUEC12470	M	Serra da Bodoquena, Mato Grosso do Sul, Brazil	Yes	-	This work
D. jimi	ZUEC13468	M	Botucatu, São Paulo, Brazil, Brazil	-	JX287413	This work
D. jimi	ZUEC13469	M	Botucatu, São Paulo, Brazil	-	JX287414	This work
D. jimi	ZUEC13470	M	Botucatu, São Paulo, Brazil	-	JX287415	This work
D. jimi	ZUEC12401	M	Uberlândia, Minas Gerais, Brazil	Yes	-	This work
D. jimi	ZUEC12402	M	Uberlândia, Minas Gerais, Brazil	Yes	-	This work
D. jimi	ZUEC12403	M	Uberlândia, Minas Gerais, Brazil	Yes	-	This work
D. jimi	ZUEC12404	M	Uberlândia, Minas Gerais, Brazil	Yes	JX287416	This work
D. jimi	ZUEC12405	M	Uberlândia, Minas Gerais, Brazil	Yes	-	This work
D. jimi	ZUEC12406	M	Uberlândia, Minas Gerais, Brazil	Yes	JX287417	This work
D. jimi	ZUEC12407	M	Uberlândia, Minas Gerais, Brazil	Yes	JX287418	This work
D. jimi	ZUEC12408	M	Uberlândia, Minas Gerais, Brazil	Yes	-	This work
D. jimi	ZUEC12463	M	Uberlândia, Minas Gerais, Brazil	Yes	-	This work
D. jimi	ZUEC12464	M	Uberlândia, Minas Gerais, Brazil	Yes	-	This work
D. jimi	ZUEC12305	M	Uberlândia, Minas Gerais, Brazil	Yes	-	This work
D. jimi	ZUEC12306	M	Uberlândia, Minas Gerais, Brazil	Yes	-	This work
D. jimi	ZUEC12307	M	Uberlândia, Minas Gerais, Brazil	Yes	-	This work
D. jimi	ZUEC12308	M	Uberlândia, Minas Gerais, Brazil	Yes	-	This work
D. jimi	ZUEC12309	M	Uberlândia, Minas Gerais, Brazil	Yes	-	This work
D. jimi	ZUEC12310	M	Uberlândia, Minas Gerais, Brazil	Yes	-	This work
D. leali	KU 215259	-	Cuzco Amazonico, Madre de Dios, Peru	-	AY819451	[28] Wiens et al. (2005)
D. microcephalus	UTA 50632	-	Atlantida, Honduras	-	AY819371	[28] Wiens et al. (2005)
D. microcephalus	UTA A-50632	-	Atlantida, Honduras	-	AY843643	[1] Faivovich et al. (2005)
D. microcephalus	MVZ203881	-	Guanacaste, Costa Rica	-	EF566945	Unpublished
D. minusculus	-	-	-	-	EF376025	Unpublished
D. minutus	ZUEC12409	M	Serra da Bodoquena, Mato Grosso do Sul, Brazil	-	JX287424	This work
D. minutus	ZUEC12410	M	Serra da Bodoquena, Mato Grosso do Sul, Brazil	-	JX287425	This work
D. minutus	ZUEC12411	M	Serra da Bodoquena, Mato Grosso do Sul, Brazil	-	JX287426	This work
D. minutus	ZUEC13191	M	Campinas, São Paulo, Brazil	-	JX287421	This work
D. minutus	ZUEC13193	M	Campinas, São Paulo,Brazil	-	JX287422	This work
D. minutus	ZUEC18133	M	Campinas, São Paulo, Brazil	-	JX287423	This work
D. minutus	ZUEC12414	M	Nova Itapirema, São Paulo, Brazil	-	JX287419	This work
D. minutus	ZUEC12415	M	Nova Itapirema, São Paulo, Brazil	-	JX287420	This work
D. minutus	SMRP171.1	M	Telêmaco Borba, Paraná, Brazil	-	JX287429	This work
D. minutus	SMRP171.2	M	Telêmaco Borba, Paraná, Brazil	-	JX287428	This work
D. minutus	SMRP171.3	M	Telêmaco Borba, Paraná, Brazil	-	JX287427	This work
D. minutus	DZSJRP7970	M	Vitória Brasil, São Paulo, Brazil	-	JX287430	This work
D. minutus	DZSJRP7975	M	Vitória Brasil, São Paulo, Brazil	-	JX287431	This work
D. minutus	DZSJRP7976	M	Vitória Brasil, São Paulo, Brazil	-	JX287432	This work
D. nanus	SMRP47.2	-	Nova Itapirema, São Paulo, Brazil	-	JX287443	This work
D. nanus	SMRP47.3	-	Nova Itapirema, São Paulo, Brazil	-	JX287444	This work
D. nanus	SMRP47.4	-	Nova Itapirema, São Paulo, Brazil	-	JX287445	This work
D. nanus	ZUEC11416	-	Nova Itapirema, São Paulo, Brazil	-	JX287446	This work
D. nanus	SMRP47.12	M	Nova Itapirema, São Paulo, Brazil	-	JX287447	This work
D. nanus	SMRP47.20	M	Nova Itapirema, São Paulo, Brazil	-	JX287448	This work
D. nanus	ZUEC12261	M	Botucatu, São Paulo, Brazil	Yes	JX287438	This work
D. nanus	ZUEC12265	M	Botucatu, São Paulo, Brazil	Yes	JX287439	This work
D. nanus	ZUEC12392	F	Serra da Bodoquena, Mato Grosso do Sul, Brazil		JX287475	This work
D. nanus	ZUEC12393	F	Serra da Bodoquena, Mato Grosso do Sul, Brazil		JX287474	This work
D. nanus	ZUEC13179	M	Serra da Bodoquena, Mato Grosso do Sul, Brazil	Yes	JX287476	This work
D. nanus	ZUEC13180	M	Serra da Bodoquena, Mato Grosso do Sul, Brazil	Yes	JX287477	This work
D. nanus	ZUEC11899	F	Corumbá, Mato Grosso do Sul, Brazil	-	JX287436	This work
D. nanus	ZUEC11904	M	Corumbá, Mato Grosso do Sul, Brazil	-	JX287437	This work
D. nanus	ZUEC11879	M	Bacabal, Maranhão, Brazil	-	JX287433	This work
D. nanus	ZUEC11886	M	Bacabal, Maranhão, Brazil	-	JX287434	This work
D. nanus	ZUEC11887	M	Bacabal, Maranhão, Brazil	-	JX287435	This work
D. nanus	ZUEC12214	?	Castanho, Amazonas, Brazil	-	JX287440	This work
D. nanus	ZUEC12215	?	Castanho, Amazonas, Brazil	-	JX287441	This work
D. nanus	ZUEC12217	?	Castanho, Amazonas, Brazil	-	JX287442	This work
D. nanus	ZUEC12382	M	Telêmaco Borba, Paraná, Brazil	Yes	-	This work
D. nanus	ZUEC12383	M	Telêmaco Borba, Paraná, Brazil	Yes	JX287449	This work
D. nanus	ZUEC12384	M	Telêmaco Borba, Paraná, Brazil	Yes	JX287450	This work
D. nanus	ZUEC12385	M	Telêmaco Borba, Paraná, Brazil	Yes	-	This work
D. nanus	ZUEC12386	M	Telêmaco Borba, Paraná, Brazil	Yes	-	This work
D. nanus	ZUEC12387	M	Telêmaco Borba, Paraná, Brazil	Yes	-	This work
D. nanus	ZUEC12388	M	Telêmaco Borba, Paraná, Brazil	Yes	-	This work
D. nanus	ZUEC12389	M	Telêmaco Borba, Paraná, Brazil	Yes	-	This work
D. nanus	ZUEC12390	F	Telêmaco Borba, Paraná, Brazil	Yes	JX287451	This work
D. nanus	ZUEC12391	M	Telêmaco Borba, Paraná, Brazil	Yes	-	This work
D. nanus	USNM-Field Number 53122		South of Luiz Antonio, São Paulo, Brazil	-	AY819373	[28] Wiens et al. (2005)
D. nanus	MACN 37785		Dto. Islas del Ibicuy, Entre Rios, Argentina	-	AY549346	[30] Faivovich et al. (2004)
D. nanus	-	-	-	-	EF376026^a^	Unpublished
D. rhodopeplus	MHZ 462		Loreto, Peru	-	AY843658	[1] Faivovich et al. (2005)
D. rhodopeplus	KU 221906		Loreto, Peru	-	DQ380371	[31] Wiens et al. (2006)
D. robertmertensi	MZFC 15824		Oaxaca, Mexico	-	AY819452	[28] Wiens et al. (2005)
D. rubicundulus^b^	IT-H 0653		Buri, São Paulo, Brazil	-	AY843661	[1] Faivovich et al. (2005)
D. sanborni	SMRP48.23	M	Nova Itapirema, São Paulo, Brazil	-	JX287459	This work
D. sanborni	SMRP48.25	M	Nova Itapirema, São Paulo, Brazil	-	JX287460	This work
D. sanborni	SMRP48.26	M	Nova Itapirema, São Paulo, Brazil	-	JX287461	This work
D. sanborni	ZUEC12416	M	Salesópolis, São Paulo, Brazil	Yes	JX287462	This work
D. sanborni	ZUEC12417	M	Salesópolis, São Paulo, Brazil	Yes	JX287463	This work
D. sanborni	ZUEC12419	M	Salesópolis, São Paulo, Brazil	Yes	JX287464	This work
D. sanborni	ZUEC12433	M	Botucatu, São Paulo, Brazil	Yes	JX287465	This work
D. sanborni	ZUEC12434	M	Botucatu, São Paulo, Brazil	Yes		This work
D. sanborni	ZUEC12435	M	Botucatu, São Paulo, Brazil	Yes		This work
D. sanborni	ZUEC12436	M	Botucatu, São Paulo, Brazil	Yes		This work
D. sanborni	ZUEC12437	M	Botucatu, São Paulo, Brazil	Yes		This work
D. sanborni	ZUEC12438	M	Botucatu, São Paulo, Brazil	Yes	JX287466	This work
D. sanborni	ZUEC12439	F	Botucatu, São Paulo, Brazil	Yes		This work
D. sanborni	ZUEC12440	M	Botucatu, São Paulo, Brazil	Yes		This work
D. sanborni	ZUEC12441	M	Botucatu, São Paulo, Brazil	Yes		This work
D. sanborni	ZUEC12442	M	Botucatu, São Paulo, Brazil	Yes		This work
D. sanborni	ZUEC12443	M	Botucatu, São Paulo, Brazil	Yes	JX287467	This work
D. sanborni	ZUEC12444	M	Botucatu, São Paulo, Brazil	Yes		This work
D. sanborni	ZUEC12445	M	Botucatu, São Paulo, Brazil	Yes		This work
D. sanborni	ZUEC12446	M	Botucatu, São Paulo, Brazil	Yes		This work
D. sanborni	ZUEC12447	M	Botucatu, São Paulo, Brazil	Yes		This work
D. sanborni	ZUEC12448	M	Botucatu, São Paulo, Brazil	Yes		This work
D. sanborni	ZUEC12449	M	Botucatu, São Paulo, Brazil	Yes		This work
D. sanborni	ZUEC12450	M	Botucatu, São Paulo, Brazil	Yes		This work
D. sanborni	ZUEC12451	M	Botucatu, São Paulo, Brazil	Yes		This work
D. sanborni	ZUEC12452	F	Botucatu, São Paulo, Brazil	Yes		This work
D. sanborni	ZUEC12453	M	Botucatu, São Paulo, Brazil	Yes		This work
D. sanborni	ZUEC13457	M	Torres, Rio Grande do Sul, Brazil	Yes	JX287468	This work
D. sanborni	ZUEC13460	M	Torres, Rio Grande do Sul, Brazil	Yes	JX287469	This work
D. sanborni	ZUEC13461	M	Torres, Rio Grande do Sul, Brazil	Yes	JX287470	This work
D. sanborni	MACN 38638		Dto. Islas del Ibicuy, Entre Rios, Argentina	-	AY843663	[1] Faivovich et al. (2005)
D. sartori	MZFC 16014		Guerrero, Mexico	-	AY819453	[28] Wiens et al. (2005)
D. walfordi	ZUEC12190	M	Guajará Mirim, Roraima Brazil^c^	Yes		This work
D. walfordi	ZUEC12191	M	Guajará Mirim, Roraima Brazil^c^	Yes		This work
D. walfordi	ZUEC12192	M	Guajará Mirim, Roraima Brazil^c^	Yes	JX287472	This work
D. walfordi	ZUEC12193	M	Guajará Mirim, Roraima Brazil^c^	Yes	JX287471	This work
D. walfordi	ZUEC12194	M	Guajará Mirim, Roraima Brazil^c^	Yes	JX287473	This work
D. walfordi	ZUEC12195	M	Guajará Mirim, Roraima Brazil^c^	Yes		This work
D. walfordi	ZUEC12196	M	Guajará Mirim, Roraima Brazil^c^	Yes		This work
D. walfordi	ZUEC12197	M	Guajará Mirim, Roraima Brazil^c^	Yes		This work
D. walfordi	ZUEC12198	M	Guajará Mirim, Roraima Brazil^c^	Yes		This work
D. walfordi	ZUEC12199	M	Guajará Mirim, Roraima Brazil^c^	Yes		This work
D. walfordi	ZUEC12200	M	Guajará Mirim, Roraima Brazil^c^	Yes		This work
D. walfordi	ZUEC12201	M	Guajará Mirim, Roraima Brazil^c^	Yes		This work
D. walfordi	ZUEC12202	M	Guajará Mirim, Roraima Brazil^c^	Yes		This work
D. walfordi	ZUEC12203	M	Guajará Mirim, Roraima Brazil^c^	Yes		This work
D. walfordi	ZUEC12204	M	Guajará Mirim, Roraima Brazil^c^	Yes		This work
D. walfordi	ZUEC12205	M	Guajará Mirim, Roraima Brazil^c^	Yes		This work
D. walfordi	MJH 129		Brazil	-	AY843683	[1] Faivovich et al. (2005)
D. aff. cruzi	SMRP193.2	M	Porto Nacional, Tocantins, Brazil	-	JX287398	This work
D. aff. cruzi	SMRP193.3	M	Porto Nacional, Tocantins, Brazil	-	JX287399	This work
D. aff. cruzi	SMRP193.4	M	Porto Nacional, Tocantins, Brazil	-	JX287400	This work
Other groups of Dendropsophus						
D. allenorum	KU 215190	-	Cuzco Amazonico, Madre de Dios, Peru	-	DQ380348	[31] Wiens et al. (2006)
D. anceps	CFBH 5797	-	Linhares, Espírito Santo, Brazil	-	AY843597	[1] Faivovich et al. (2005)
D. aperomeus	KU 212083	-	Rioja, San Martin, Peru	-	AY819450	[28] Wiens et al. (2005)
D. bifurcus	-	-	-	-	AY362975	Jungfer et al. (2010)
D. bifurcus	KU 217514	-	Limon, Morona-Santiago, Ecuador	-	DQ380350	[31] Wiens et al. (2006)
D. brevifrons	MJH 7101	-	Huanuco, Rio Llullapichis, Panguana, Peru	-	AY843611	[1] Faivovich et al. (2005)
D. brevifrons	WED 58779	-	Napo, Ecuador	-	DQ380351	[31] Wiens et al. (2006)
D. carnifex	KU 218300	-	Pichincha, Ecuador	-	AY819424	[28] Wiens et al. (2005)
D. carnifex	DFCH-USFQ 899	-	Pichincha, Ecuador	-	AY843616	[1] Faivovich et al. (2005)
D. ebraccatus	UTA 51789	-	Mataglpa, Comarca Penas Blancas,Finca San Sebastian, Nicaragua	-	AY819367	[28] Wiens et al. (2005)
D. ebraccatus	RdS 790	-	Stann Creek District, Belize	-	AY843624	[1] Faivovich et al. (2005)
D. elegans	LM 3135	-		-	DQ380355	[31] Wiens et al. (2006)
D. giesleri	CFBH S/N	-	Ubatuba (Picinguaba), São Paulo, Brazil	-	AY843629	[1] Faivovich et al. (2005)
D. koechlini	KU 215248	-	Cuzco Amazonico, Madre de Dios, Peru	-	AY819369	[28] Wiens et al. (2005)
D. labialis	QULC 97005	-	Parque Natural Nacional Chingaza, Colombia	-	AY843635	[1] Faivovich et al. (2005)
D. leucophyllatus	KU 215274	-	Cuzco Amazonico, Madre de Dios, Peru	-	DQ380360	[31] Wiens et al. (2006)
D. marmoratus	MJH 7116	-	Huanuco, Rio Llullapichis, Panguana, Peru	-	AY843640	[1] Faivovich et al. (2005)
D. marmoratus	USNM 317326	-	Vicinity of Huampami, Amazonas, Peru	-	AY819432	[28] Wiens et al. (2005)
D. miyatai	JPC 10772; LSUMZ H-12939	-	Sucumbios, Ecuador	-	AY843647	[1] Faivovich et al. (2005)
D. parviceps	AMNH A-139315	-	Centro Experimental da UFAC, Acre, Brazil	-	AY843652	[1] Faivovich et al. (2005)
D. parviceps	WED 50309	-		-	DQ380367	[31] Wiens et al. (2006)
D. pelidna	KU 181108	-	Betania, Tachira, Venezuela	-	AY819434	[28] Wiens et al. (2005)
D. riveroi	KU 21 7613	-	Sucumbios, Ecuador	-	DQ380372	[31] Wiens et al. (2006)
D. salli		-		-	AY362976	[32] Jungfer et al. (2010)
D. sarayacuensis	KU 221916	-	Teniente Lopez, Loreto, Peru	-	DQ380373	[31] Wiens et al. (2006)
D. schubarti	WED 57619	-		-	DQ380374	[31] Wiens et al. (2006)
D. seniculus	CFBH 5761	-	Angra dos Reis, Rio de Janeiro, Brazil	-	AY843666	[1] Faivovich et al. (2005)
D. triangulum	KU 202745	-	Misahualli, Napo, Ecuador	-	AY326053	[33] Darst & Cannatella (2004)
D. triangulum	MJH 3844	-	Lago Catalao, Acre, Brazil	-	AY843680	[1] Faivovich et al. (2005)
D. triangulum	KU 217664	-	Sucumbios, Ecuador	-	DQ380377	[31] Wiens et al. (2006)
Other genera						
Scarthyla goinorum	KU 215423	-		-	AY819389	[28] Wiens et al. (2005)
Scarthyla goinorum	QULC 2340	-	Igarape Nova Empresa, Amazonas, Brazil	-	AY843752	[1] Faivovich et al. (2005)
Scarthyla goinorum (= S. ostinodactyla)	KU 205763	-	Cuzco Amazonico, Madre de Dios, Peru	-	AY326035	[33] Darst & Cannatella (2004)
Xenohyla truncata	CFBH 7600	-	Restinda de Marica, Rio de Janeiro, Brazil	-	AY843775	[1] Faivovich et al. (2005)
Phyllodytes luteolus		-	Guarapari, Sepetiba, Brazil	-	DQ403729	Unpublished
Pseudis minuta	MACN 37786	-	Dto. Islas del Ibicuy, Entre Rios, Argentina	-	AY843739	[1] Faivovich et al. (2005)

DZSJRP: Coleção Científica Amphibia Adults, Department of Zoologia e Botânica, Universidade Estadual Paulista (UNESP), São José do Rio Preto, São Paulo, Brazil; SMRP: Collection of tissue and chromosome preparation “Shirlei Maria Recco Pimentel”, deposited at the Department of Structural and Functional Biology at the Biology Institute of the University of Campinas, Campinas, São Paulo, Brazil; ZUEC: Museu de Zoologia “Prof. Adão José Cardoso” (ZUEC), Universidade Estadual de Campinas (UNICAMP), Campinas, São Paulo, Brazil. ^a^This is a 373 bp sequence and the segment from nucleotide 2 to nucleotide 361 is exactly the same as the sequence JF973312 described by Fouquet et al. (2011). Because of the identity of these sequences and their short size, we included the sequence EF376026 but not the sequence JF973312 in our analyses.

^b^Taxonomic questions exist regarding this sequence. See the text for details.

^c^The type locality of *D. walfordi*, designated Forte Príncipe da Beira (see Frost, 2011, for references), is located in Guajará-Mirim, RO.

Identification of all the specimens employed in the cytogenetic and phylogenetic analyses, their procedence and voucher number. The GenBank accession number of each DNA sequence and the specimens which were karyotyped are indicated. DZSJRP: Coleção Científica Amphibia Adults, Department of Zoologia e Botânica, Universidade Estadual Paulista (UNESP), São José do Rio Preto, São Paulo, Brazil; SMRP: Collection of tissue and chromosome preparation “Shirlei Maria Recco Pimentel”, deposited at the Department of Structural and Functional Biology at the Biology Institute of the University of Campinas, Campinas, São Paulo, Brazil; ZUEC: Museu de Zoologia “Prof. Adão José Cardoso” (ZUEC), Universidade Estadual de Campinas (UNICAMP), Campinas, São Paulo, Brazil.

In the phylogenetic analyses, we included 12S rDNA sequences (available in GenBank) from 30 species of *Dendropsophus*, as well as sequences from *Xenohyla truncata*, *Pseudis minuta*, *Scarthyla goinorum* and *Phyllodytes luteolus*, the last of which was used as the root (Table 1). We avoided the inclusion of short partial sequences (those with less than 50% of the length of the fragment of interest). The only exception was for a sequence of *D. nanus* from French Guiana (EF376026), a locality from which we could not sample.

### Chromosomal analysis

The chromosome preparations were obtained from intestinal and testicular cell suspensions, as described by King and Rofe [34] or Schmid [35]. Prior to intestine and testes removal, the animals were deeply anesthetized. Chromosome preparations were stained with 10% Giemsa, processed for C-banding [36] and subjected to Ag-NOR staining [37] and fluorescent *in situ* hybridization (FISH) [38] with the rDNA probe HM123 [39] (except for *D. elianeae)*. All chromosome preparations were analyzed under an Olympus BX60 microscope. The chromosomes were classified as proposed by Green and Sessions [27].

### DNA extraction, amplification and sequencing

Genomic DNA was extracted from liver or muscle tissue stored at −70°C in the tissue bank of the Department of Structural and Functional Biology-UNICAMP, Campinas, SP, Brazil, using the TNES method. Tissue samples were immersed in TNES buffer solution (50 mM Tris pH 7.5, 400 mM NaCl, 20 mM EDTA, 0.5% SDS). The solution was subsequently supplemented with proteinase K (to a final concentration of 100 μg/mL), and the samples were incubated for 5 hours at 55°C. Then, 1/3 volume of NaCl 5M was added, and the samples were centrifuged. DNA was precipitated from the supernatant with isopropyl alcohol, washed with ethanol (70%), resuspended in TE (10 mM Tris–HCl, 1 mM EDTA pH 8.0) and stored at −20°C.

The mitochondrial 12S ribosomal gene was partially amplified using the primers MVZ 59(L) and MVZ 50(H) [40]. The PCR-amplified products were purified with the GFX PCR and Gel Band DNA Purification Kits (GE Healthcare, England) and directly used as templates for sequencing in an automatic ABI/Prism DNA sequencer (Applied Biosystems, Foster City, CA, USA) using the BigDye Terminator Kit (Applied Biosystems, Foster City, CA, USA), as recommended by the manufacturer. DNA sequences were bi-directionally sequenced and edited using Bioedit version 7.0.1 (http://www.mbio.ncsu.edu/BioEdit/bioedit.html).

### Phylogenetic inferences

Fragments of approximately 810 bps of the 12S ribosomal genes from 80 specimens of *Dendropsophus* were sequenced as described above, and a data matrix consisting of 133 OTUs, including five sequences from outgroup species and a total of 37 species of *Dendropsophus*, was constructed. The GenBank accession numbers for all of the sequences used are presented in Table 1. Because parsimony [1] and likelihood [12,13] criteria have been employed for the phylogenetic studies of *Dendropsophus*, we conducted both types of analyses. When using parsimony criterion, phylogenetic relationships were inferred (i) from analyses under dynamic homology, as implemented in the software POY v.4.1.2.1 [41], or (ii) from aligned sequences using the software TNT v.1.1 [42]. A Bayesian analysis was implemented in the software MrBayes v.3.1.2 [43] using the model GTR + I + G, inferred with the software MrModeltest v.2.3 [44]. For the analyses using TNT and MrBayes, the sequences were first aligned with Clustal W [45], and a matrix was generated with 852 characters.

The phylogenetic searches performed with POY included tree building (of Wagner trees), tree bisection–reconnection (TBR) swapping, perturbation using a parsimony ratchet and tree fusing. The analyses were run with a maximum execution time of 48 h and an opening indel cost of 3, indel extension cost of 1 and nucleotide substitution cost of 1 using the command “transform (tcm:(1, 1), gap_opening:2)”. To obtain an implied alignment from the POY analysis, the characters were transformed into static characters, and the generated matrix was exported using the command “phastwinclad.” The exported matrix was loaded with TNT v.1.1 to calculate the bootstrap support based on 1,000 pseudoreplicates.

For the phylogenetic analysis using TNT software, the most parsimonious trees were inferred through heuristic searches performed using the command *xmult*, which combined sectorial searches, the ratchet, tree drifting and tree fusing. Gaps were considered to be missing data. The bootstrap values of the branches inferred in this analysis were calculated with 1000 pseudoreplicates.

For the Bayesian inferences, two simultaneous analyses were run, each with four chains (three heated and one cold). In each analysis, 2,980,000 generations were run and one tree was sampled every 100 generations. A consensus topology and the posterior probability for each node were produced after discarding the first 25% of the trees generated. The ASDSF (Average Standard Deviation of Split Frequencies) value was below 0.01, and the PSRF (Potential Scale Reduction Factor) values were approximately 1.000.

## Competing interests

The authors declare that they have no competing interests.

## Authors’ contributions

LRM acquired the cytogenetic data and helped draft the manuscript. GTBTE acquired most of the DNA sequences. LBL acquired some of the cytogenetic data and DNA sequences, conducted the phylogenetic analyses and helped draft the manuscript. DCRF, APL, GVA and AAG helped collect and identified the specimens, provided support on zoological information and revised the manuscript. SMRP designed and coordinated the study and revised the manuscript. All authors read and approved the final manuscript.

## Supplementary Material

Additional file 1: Figure S1 Strict consensus cladogram of four most parsimonious trees scored at 2195 inferred from TNT analyses of 12S rDNA sequences. Numbers adjacent to nodes indicate bootstrap values. The karyotype fundamental number (FN) is indicated for some species. The asterisk indicates the node of the clade that includes the species of the *D. microcephalus*  group. Click here for file

Additional file 2: Figure S2 Topology inferred from Bayesian analysis of 12S rDNA sequences. Numbers adjacent to nodes indicate posterior probabilities. The asterisk indicates the node of the clade that includes the species of the *D. microcephalus*  group. Click here for file

Additional file 3: Figure S3 Karyotype of the ZUEC 13179 specimen of *D. nanus* with FN = 53. In A, Giemsa-stained karyotype arranged from the same metaphase which is shown in **B** after silver staining. Note the heteromorphic pair 6. Click here for file
