# Supplementary figures and images for: Comparative cytogenetic analysis of some species of the Dendropsophus microcephalus group (Anura, Hylidae) in the light of phylogenetic inferences
Source: BMC Genet. 2013 Jul 3;14:59. doi: 10.1186/1471-2156-14-59 (PMC3710474; doi:10.1186/1471-2156-14-59)

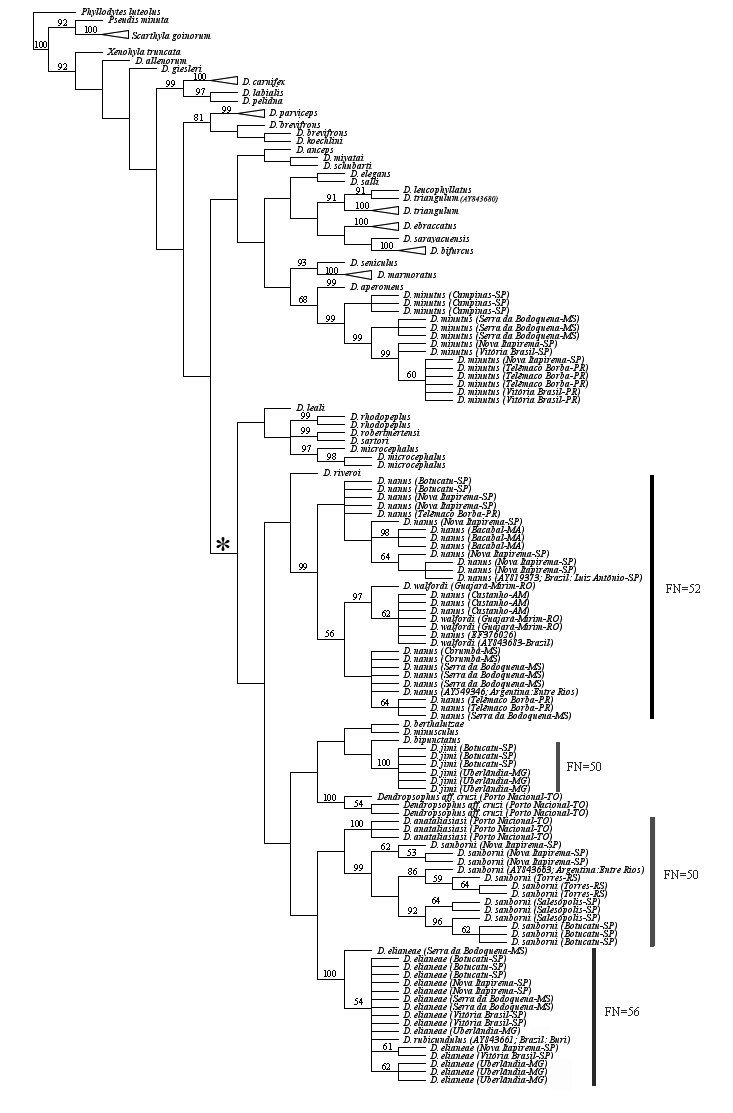

Supplement: Additional file 1: Figure S1 — Strict consensus cladogram of four most parsimonious trees scored at 2195 inferred from TNT analyses of 12S rDNA sequences. Numbers adjacent to nodes indicate bootstrap values. The karyotype fundamental number (FN) is indicated for some species. The asterisk indicates the node of the clade that includes the species of the D. microcephalus group. [file 1471-2156-14-59-S1.tiff]

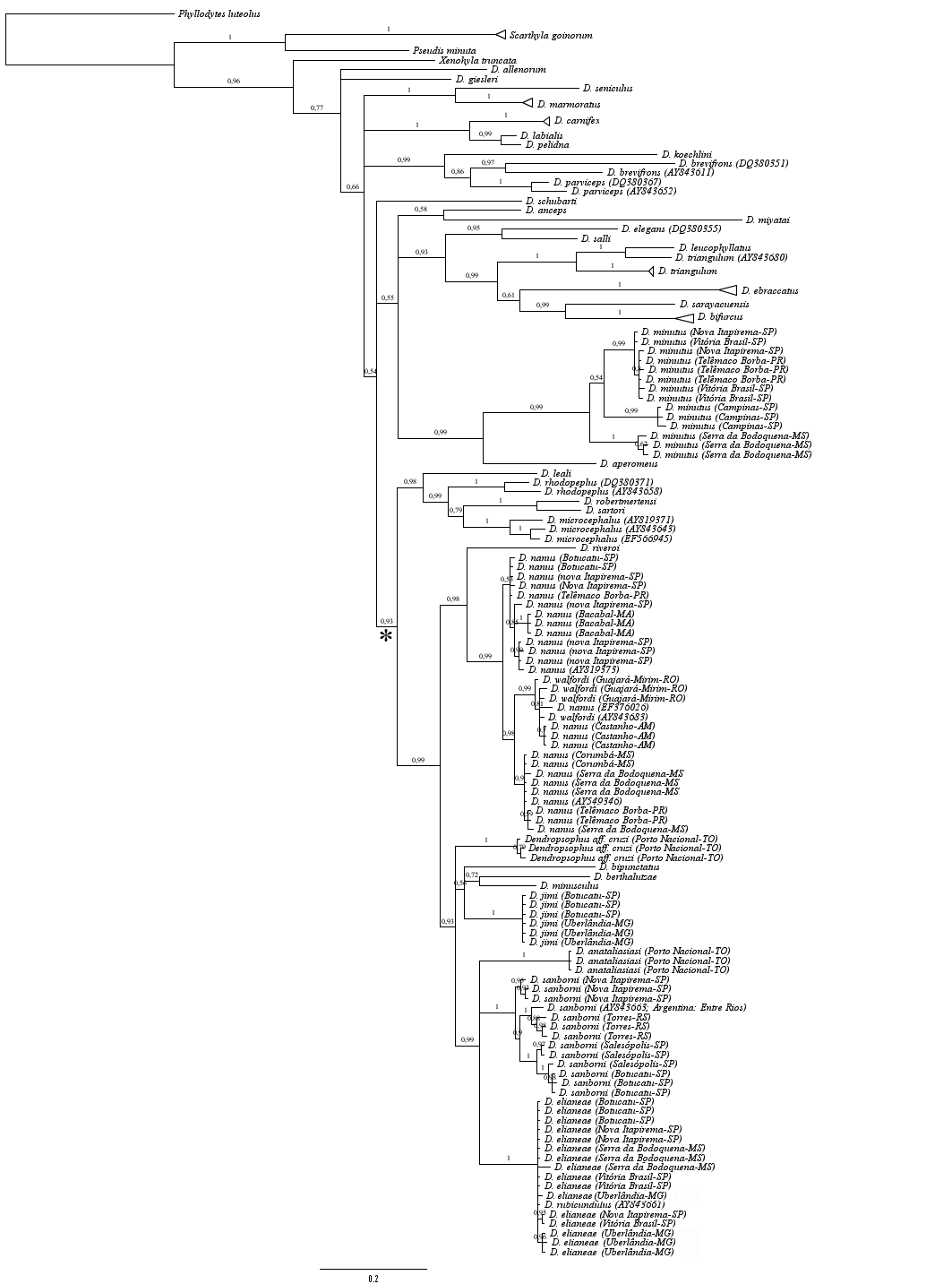

Supplement: Additional file 2: Figure S2 — Topology inferred from Bayesian analysis of 12S rDNA sequences. Numbers adjacent to nodes indicate posterior probabilities. The asterisk indicates the node of the clade that includes the species of the D. microcephalus group. [file 1471-2156-14-59-S2.tiff]

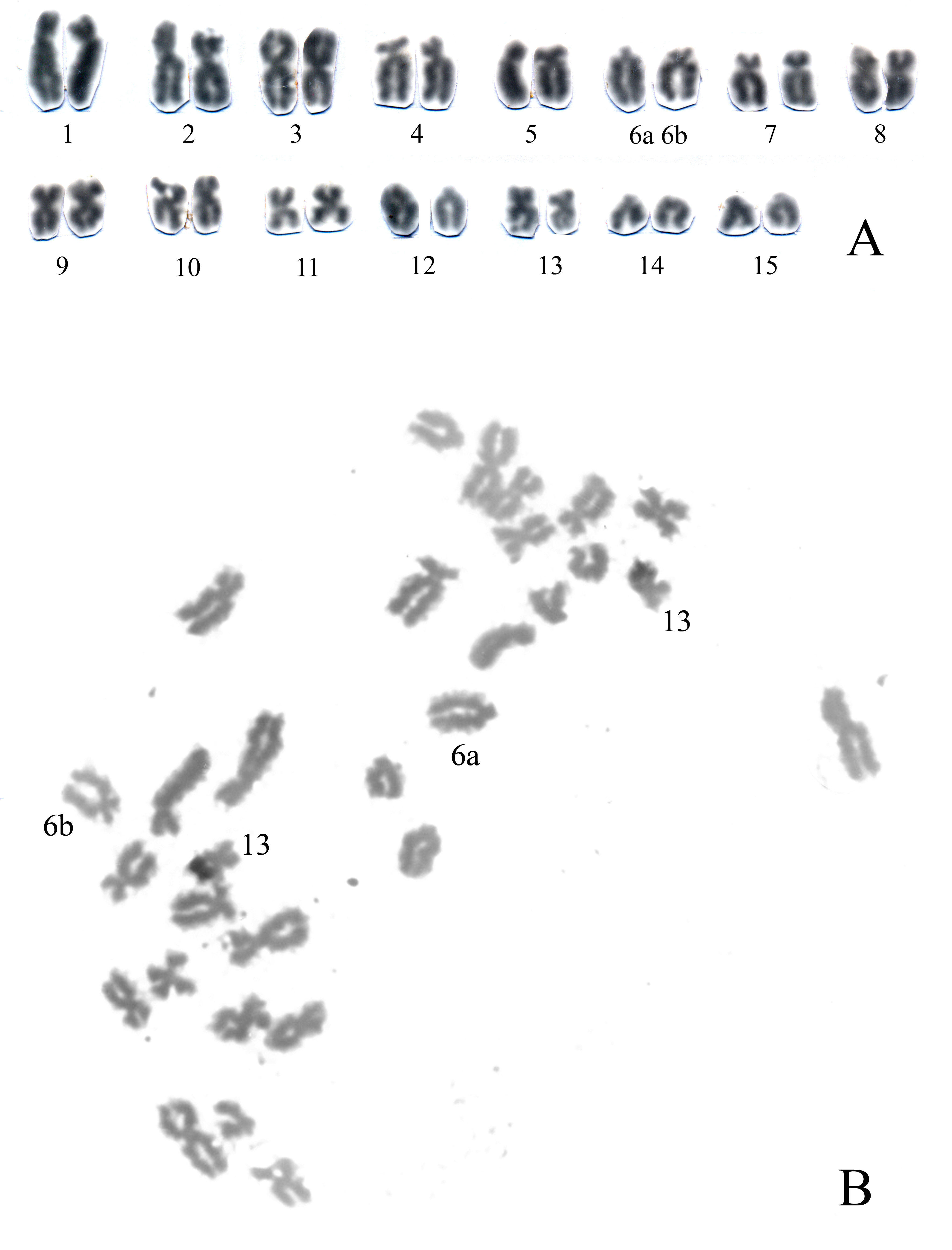

Supplement: Additional file 3: Figure S3 — Karyotype of the ZUEC 13179 specimen of D. nanus with FN = 53. In A, Giemsa-stained karyotype arranged from the same metaphase which is shown in B after silver staining. Note the heteromorphic pair 6. [file 1471-2156-14-59-S3.tiff]
